# Extremely Low Frequency Magnetic Field Exposure and Parkinson’s Disease—A Systematic Review and Meta-Analysis of the Data

**DOI:** 10.3390/ijerph120707348

**Published:** 2015-06-30

**Authors:** Anke Huss, Tom Koeman, Hans Kromhout, Roel Vermeulen

**Affiliations:** 1 Institute for Risk Assessment Sciences, Utrecht University, Utrecht 3584CM, The Netherlands; E-Mails: t.koeman@uu.nl (T.K.); h.kromhout@uu.nl (H.K.); r.c.h.vermeulen@uu.nl (R.V.); 2 Institute of Social and Preventive Medicine, University of Bern, Bern 3012, Switzerland; 3 Julius Centre for Public Health Sciences and Primary Care, University Medical Centre, Utrecht 3584CG, The Netherlands

**Keywords:** meta-analysis, Parkinson’s disease, magnetic field, human

## Abstract

*Objective*: To examine the association between occupational exposure to extremely-low-frequency magnetic fields (ELF-MF) and Parkinson’s disease. *Methods*: We systematically searched publications reporting risk estimates of Parkinson’s disease in workers exposed to ELF-MF. Summary relative risks were obtained with random effects meta-analysis. *Results*: We included 11 studies. To assign exposure, four studies evaluated occupational records, four used census, interview or questionnaire information and three used death certificates. Risk of Parkinson’s disease was not elevated in workers exposed to ELF-MF with a summary relative risk of 1.05, 95% CI 0.98–1.13. *Conclusions*: Overall, there was no evidence that the exposure to ELF-MF increases the risk of Parkinson’s disease.

## 1. Introduction

Since the majority of Parkinson’s disease cases are thought to be sporadic, environmental factors may play an important role in the development of the disease [1,2]. One of these environmental factors to be potentially associated with Parkinson’s disease is the exposure to extremely-low-frequency magnetic fields (ELF-MF). Since many workers are exposed to ELF-MF above general background levels, this exposure has a potentially strong impact on public health even if the risks to the individual are low. Studies have evaluated both residential [3] as well as occupational [4,5,6,7,8,9,10,11,12] exposure to ELF-MF. A previous systematic review published in 2006 [13], reviewing papers [4,5,6,7,8], concluded that few studies had shown an association between magnetic field exposure and Parkinson’s disease. A recent review did not provide quantitative summaries of the published studies [14]. Since the latest review, several new studies have been published [15,16,17,18]. We updated the study base and performed a systematic review and meta-analysis on studies analyzing the effect of occupational ELF-MF on Parkinson’s disease.

## 2. Methods

We searched publications in EMBASE and MEDLINE using the search words “neurodegenerative”, “parkinson”, in combination with “electromagnetic”, “electric”, “magnetic”, “EMF”, “electrical”, and “occupational”, “occupation”, “job”, “work”, “workplace”, “worker”, as well as “exposure” or “exposed”. We additionally checked a specialist literature database, using “Parkinson” as search term [19]. We included peer-reviewed papers published in English language until 9 March 2015 if they reported risk estimates of Parkinson’s disease in association with ELF-MF exposure. We excluded studies that did not provide estimates of magnetic field exposures.

If risk estimates were presented for more than two ELF-MF exposure levels (e.g., medium exposure *versus* lowest, and high *versus* lowest), we pooled risk estimates across all presented exposure categories (except the reference group) to obtain a comparison of “higher *versus* lowest” exposure, using a within-study meta-analysis. In addition, we extracted risk estimates of the highest (cumulative) reported EMF exposure category (“highest-longest *versus* lowest”). If both were presented, we preferred adjusted risk estimates over unadjusted ones. If the outcome was assessed from death certificates and presented as primary cause of death or as listed anywhere on the death certificate, we extracted the latter for our analysis. Of a publication on an industrial cohort we used risk estimates reporting on morbidity [6] instead of mortality [20] and of another one the most recent update [17] instead of an earlier publication [12]. Summary risk estimates were obtained with a random effects meta-analysis [21], and an I^2^ value was calculated, which gives an indication of heterogeneity between the studies [22]. We also checked funnel plot asymmetry using the Egger test [23].

Type of exposure assessment could be related to heterogeneity between study results. In particular, study results could differ depending on whether a complete occupational history was evaluated, or if exposure to ELF-MF was assessed at only one or two points in time (e.g., when using census information). Also, there is consensus that occupations recorded on death certificates are not accurate enough to correctly assign exposure to ELF-MF [24]. We therefore used meta-regression to assess whether type of exposure assessment (job titles from occupational records evaluating the full occupational history, from censuses/questionnaires, or the longest held occupation as stated on death certificates) or the type of population (industrial cohort *versus* general population) was related to heterogeneity between study results. Because Parkinson’s disease is not in itself a fatal disease and will therefore only be registered incompletely on death certificates, we additionally evaluated if results of studies differed depending on whether Parkinson’s disease was assessed from clinical records or from death certificates.

Given the small number of studies, study characteristics were tested one at a time in separate models. All analyses were performed in Stata version 12 (StataCorp, College Station, TX, USA) with the metan, metareg, metafunnel and metabias commands.

## 3. Results

We screened 177 unique abstracts resulting from our EMBASE and MEDLINE search. We excluded 166 studies for various reasons (81 not about ELF-MF as exposure, 25 not about Parkinson’s disease, 18 mechanistic studies, 14 therapeutic studies, 24 reviews, two articles not on occupational exposure and two articles that were updated in later studies) and included 11 studies into our meta-analysis. Study characteristics are given in Table 1.

Job titles were assessed from occupational records [6,9,11,17], from censuses [4,5], from questionnaires evaluating the full occupational history [15,18], or from the longest held occupation as stated on death certificates [7,8,10]. Parkinson’s disease was either assessed by hospital records [6,15], or death certificates (International Classification of Disease, versions 8 to 10 (ICD-8/9/10), using codes ICD-8 342, ICD-9 332 and ICD-10 G20 (also G21 and G25.9 in Röösli *et al*. [9]).

**Figure 1:**
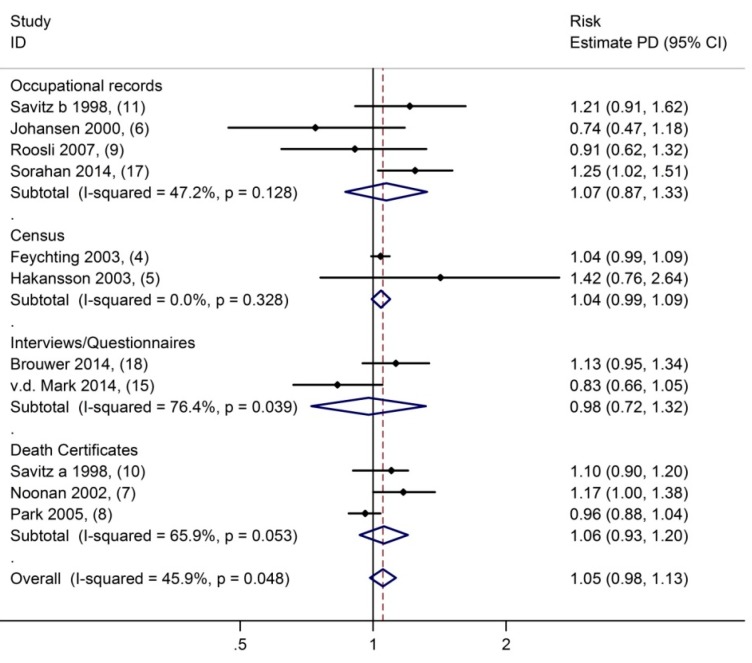
Parkinson’s disease in association with occupational exposure to extremely-low-frequency magnetic fields. Comparing higher to lowest exposure to extremely-low-frequency magnetic fields (Numbers in brackets pertain to references).

Studies reporting on the association between occupational ELF-MF exposures and Parkinson’s disease are shown in Figure 1. Heterogeneity between studies was moderate with 46%. Overall, there was no evidence that the exposure to ELF-MF was associated with Parkinson disease, the summary relative risk (sRR) was 1.05 (95% confidence interval (CI) 0.98–1.13).

**Figure 2:**
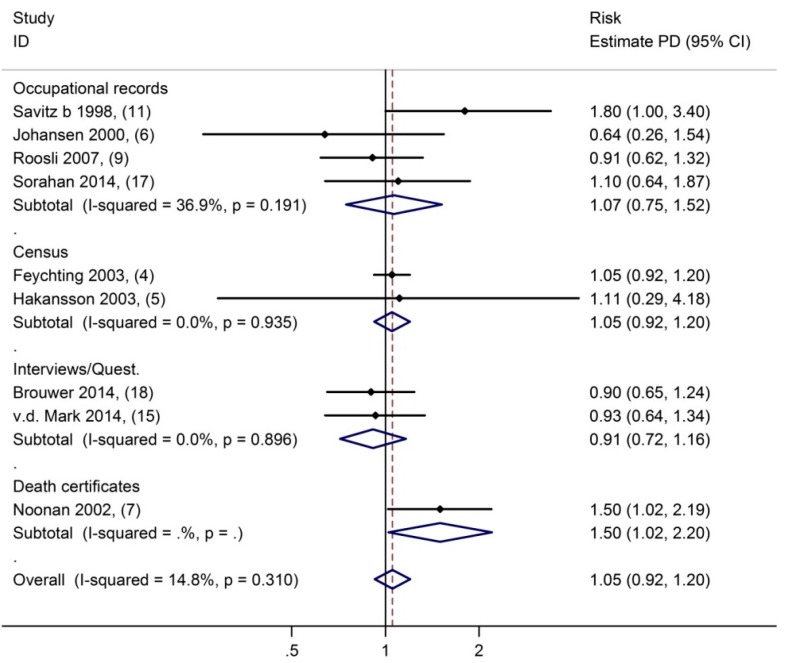
Parkinson’s disease in association with occupational exposure to extemely-low-frequency magnetic fields. Comparing highest-longest to lowest exposure to extremely-low-frequency magnetic fields (Numbers in brackets pertain to references).

Heterogeneity between studies was not explained by the type of exposure assessment (see Figure 1) or whether the study was a general population study or an industrial cohort. There were only two studies [6,15] that used clinical records to identify Parkinson’s disease cases rather than mortality but these two studies also provided no evidence for an increased risk of Parkinson’s disease in exposed workers; the sRR was 0.81 (95% C.I. 0.67–1.00). Accounting for the type of outcome assessment slightly reduced heterogeneity between studies (I^2^ = 30%). Nine studies reported risk estimates of highest-longest exposure, which resulted in a sRR of 1.05 (95% C.I. 0.92–1.20, see Figure 2). Finally, funnel plots were not indicative of funnel plot asymmetry (*p*-value from Egger test = 0.7).

**Table 1:** Characteristics of studies of occupational ELF-MF exposure and Parkinson’s disease.

Study	Design	Outcome: Source of Information	Population	Exposure	Exposure Information Source	Time Point of Exposure Assessment	Number of Cases
Savitz 1998 a [10]	Case-control	Death certificates	Deceased that had occupational information on death certificate from 25 USA states	Electrical occupation	Death certificates	Primary occupation	161
Savitz 1998 b [11]	cohort	Death certificates	Electric utility workers	ELF-MF	Occupational records	Occupational history	117
Johansen 2000 [6] ^a^	Cohort	Hospital records	Utility companies	ELF-MF	Occupational records	Occupation at baseline/census	68
Noonan 2002 [7] ^a^	Case-control	Death certificates	Deceased aged at least 60 years from Colorado, USA.	ELF-MF, electrical occupations	Death certificates	Primary occupation	1477
Feychting 2003 [4] ^a^	Cohort	Death certificates	Economically active Swedish population at census	ELF-MF	Census	Occupation at baseline/census	6101
Hakansson 2003 [5] ^a^	Cohort	Death certificates	Industry cohort of engineering workers	ELF-MF	Census	Occupation at baseline/census	45
Park 2005 [8] ^a^	Case-control	Death certificates	Deceased from 22 USA states	ELF-MF, occupation	Death certificates	Primary occupation	33,678
Röösli 2007 [9]	Cohort	Death certificates	Swiss railway employees	ELF-MF	Occupational records	Occupational history	118
Sorahan 2007 [12]	Cohort	Death certificates	Electricity generation and transmission workers	ELF-MF	Occupational and location information with modelled exposure	Occupational history	278
Sorahan 2014 [17]							
v.d. Mark 2014 [15]	Case-control	Hospital records	General population	ELF-MF, electric shocks	Interviews	Occupational history	444
Brouwer 2015 [18]	Cohort	Death certificates	General population cohort in the Netherlands	ELF-MF	Questionnaire on occupational history	Occupational history	609

Abbreviation: ELF-MF: extremely-low frequency magnetic fields; ^a^ Reviewed in earlier systematic review by Hug *et al.* [13].

## 4. Discussion

In our meta-analysis we did not identify elevated risks of Parkinson’s disease in workers exposed to ELF-MF. Two previous studies that evaluated risks in persons living in close proximity to overhead power lines also found no association with Parkinson’s disease [3,16].

Exposure misclassification is of concern in nearly all presented studies, where a variety of methods was used to assign exposure levels to job titles. For example, some studies assigned exposures based on full occupational histories, while others used occupations a person had held at one time point, such as the primary job as reported on death certificates. Within those studies that did not capture the full occupational history, the question arises in how far all relevant ELF-MF occupational exposures during the life course were evaluated. For example, a population-based study in Swedish twins asked for both the longest held job and the last occupation, and found that 31%–36% of the population reported different occupations for primary and last job [25]. A similar percentage of job changes was reported in a Swedish region between the censuses of 1960 and 1970 [26]. However, sRR were not materially different across studies that had applied different methods of exposure assessment. Several studies evaluated exposure response associations. If ELF-MF exposure was associated with Parkinson’s disease, then in principle one would expect to observe higher risks among the workers with the highest or longest exposure. This however, was not the case, sRR were equal when analyzed across exposed *versus* subjects exposed to background-levels or highest exposure category *versus* subjects exposed to background-levels.

More recently, risk of electric shocks at work has received more attention because it has been hypothesized that such shocks could be associated with the development of Amyotrophic Lateral Sclerosis (ALS) [20]. ALS is a neurodegenerative disease that has been associated with working in so-called “electrical occupations” [27]. Risk of experiencing electric shocks has been reported to be correlated to magnetic field exposures and given that they occur by accident, potential risks arising from electric shocks are more difficult to investigate. Over the last few years, job exposure matrices were developed that identified occupations in which workers are at higher risk of electric shock at work, using registered occupational electrical injuries [28,29]. The two studies that applied one of these electric shock JEMs to their data base, however, did not observe elevated risks of Parkinson’s disease in exposed workers [15,18].

By far the majority of studies relied on reporting of the outcome on death certificates, where Parkinson’s disease would be expected to be underreported. Underreporting as such would primarily lead to a loss of power in the analysis. Bias would arise if this underreporting was associated with levels of exposure to ELF-MF or if the reported causes of death include false positives. Our study indeed provided evidence that results differed depending on whether the outcome was assessed from death certificates or not. However, assessing Parkinson’s disease from clinical records also provided no evidence of increased risks. Finally, funnel plot asymmetry provided no evidence of small study effects.

## 5. Conclusions

In conclusion, studies so far do not indicate that workers exposed to magnetic fields are at higher risk of Parkinson’s disease. This is reassuring given the ubiquity of the exposure in modern life.
